# Immunohistochemical study of human embryonic brain choroid plexuses and Subcommissural Organ

**DOI:** 10.1186/1743-8454-7-S1-S47

**Published:** 2010-12-15

**Authors:** Emilia M Carmona-Calero, Ibrahim González-Marrero, Juan M González-Toledo, Agustín Castañeyra-Ruiz, Leandro Castañeyra-Ruiz, Héctor de Paz-Carmona, Isabel Hernández-Garde, Agustín Castañeyra-Perdomo

**Affiliations:** 1 Departamento de Anatomía, Facultad de Medicina, Universidad de La Laguna, Tenerife, Spain; 2 Departamento de Biotecnología, Instituto de Investigación y Ciencias de Puerto del Rosario, Fuerteventura, Spain

## Background

The choroid plexus is mainly involved in the production of cerebrospinal fluid (CSF) by using the free access to the blood compartment of the leaky vessels. In order to separate blood and CSF compartments, choroid plexus epithelial cells and tanycytes of circumventricular organs constitute the blood–CSF–brain barrier. The choroid plexus is involved in a variety of neurological disorders, including neurodegenerative, inflammatory, infectious, traumatic, neoplastic, and systemic diseases. Ab and Biondi ring tangles accumulate in the Alzheimer’s disease choroid plexus, Hartwig Wolburg and Werner Paulus (2010). The choroid plexus epithelium constitutes the structural basis of the blood-cerebrospinal fluid barrier which is important for maintaining an optimal homeostatic environment for the brain. We immunohistochemically investigated the expression of the proliferation cell nuclear antigen (PCNA), p73, TTR and caspase in the choroid plexus and the SCO.

## Materials and methods

Brains from 10 to 30 weeks of gestation (WG), from the collection of the Department of Anatomy of the University of La Laguna, were used. Brains were processed using the following standardized form: fixation in formaldehyde, post fixation in Bouin for 24 hours, dehydration and paraffin embedding, and were then cut in three (A, B, C and D) coronal and sagittal sections 10 microns thick. The A series were stained with Klüver-Barrera, B, C and D series were immunohistochemically processed using p73 (1:1000), TTR(1:400), PCNA (1:15,000) and caspase (1:200) as primary antibodies.

## Results

We observed that choroid plexus epithelial cells and tanycytes of circumventricular organs presented immunohistochemical changes. Pro-apoptotic p73 protein was detected in all parts of the SCO throughout the investigated period. TTR (pre-albumin) was found in the basal and apical process and in the secretory granules located in the ventricular cell pole. The antibodies to proliferating cell nuclear antigen (PCNA) were observed in the choroid plexus and the SCO. The immunoreactive material was located in the nuclei forming condensations in both the ependymal and hypendymal layer.

## Conclusions

These proteins are detected in the SCO and the choroid plexuses suggesting that their expression is related with secretion of CSF by the choroid plexus and the development subcommissural organ.

